# Decoding the ribosome's hidden language: rRNA modifications as key players in cancer dynamics and targeted therapies

**DOI:** 10.1002/ctm2.1705

**Published:** 2024-05-26

**Authors:** Li Cui, Jiarong Zheng, Yunfan Lin, Pei Lin, Ye Lu, Yucheng Zheng, Bing Guo, Xinyuan Zhao

**Affiliations:** ^1^ Stomatological Hospital, School of Stomatology Southern Medical University Guangzhou Guangdong China; ^2^ Division of Oral Biology and Medicine, School of Dentistry University of California, Los Angeles Los Angeles USA; ^3^ Department of Dentistry, The First Affiliated Hospital Sun Yat‐Sen University Guangzhou China

**Keywords:** cancer biology, rRNA modification, therapeutic potential

## Abstract

Ribosomal RNA (rRNA) modifications, essential components of ribosome structure and function, significantly impact cellular proteomics and cancer biology. These chemical modifications transcend structural roles, critically shaping ribosome functionality and influencing cellular protein profiles. In this review, the mechanisms by which rRNA modifications regulate both rRNA functions and broader cellular physiological processes are critically discussed. Importantly, by altering the translational output, rRNA modifications can shift the cellular equilibrium towards oncogenesis, thus playing a key role in cancer development and progression. Moreover, a special focus is placed on the functions of mitochondrial rRNA modifications and their aberrant expression in cancer, an area with profound implications yet largely uncharted. Dysregulation in these modifications can lead to metabolic dysfunction and apoptosis resistance, hallmark traits of cancer cells. Furthermore, the current challenges and future perspectives in targeting rRNA modifications are highlighted as a therapeutic approach for cancer treatment. In conclusion, rRNA modifications represent a frontier in cancer research, offering novel insights and therapeutic possibilities. Understanding and harnessing these modifications can pave the way for breakthroughs in cancer treatment, potentially transforming the approach to combating this complex disease.

## Graphical abstract

1. rRNA modifications crucially influence ribosome function and cancer development, altering cellular protein synthesis and oncogenesis.
2. Mitochondrial rRNA modifications impact cancer cell traits, including metabolic dysfunction and resistance to apoptosis.
3. Targeting rRNA modifications presents potential therapeutic avenues in cancer treatment, posing challenges and opportunities for breakthroughs.

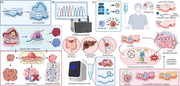

## 1 INTRODUCTION

The intricate landscape of rRNA modifications represents a pivotal aspect of post‐transcriptional regulation, playing a crucial role in the maintenance of cellular homeostasis and gene expression.^1^, ^2^ These modifications, encompassing a diverse array of chemical alterations such as methylation, pseudouridylation, and acetylation, are fundamental to the structural integrity and functional efficiency of the ribosome.^3^, ^4^, ^5^ They are intricately involved in fine‐tuning the translational machinery, ensuring the fidelity and efficiency of protein synthesis, which is vital for the proper execution of cellular processes such as response to stress, cellular differentiation, and maintenance of genomic integrity.^6^

In the context of human diseases, particularly cancer, deregulation of rRNA modifications has emerged as a significant factor.^7^ Alterations in the patterns and levels of rRNA modifications can disrupt normal ribosome function, leading to aberrant protein synthesis that contributes to the onset and progression of cancer. For instance, specific changes in rRNA modification patterns have been linked to altered translational profiles in tumour cells, promoting the synthesis of proteins that favour oncogenic processes such as uncontrolled cell division, invasion, and metastasis.^8^, ^9^ Moreover, the dysregulation of rRNA modifications is increasingly recognised for its role in the development of drug resistance in cancer. Changes in the ribosomal landscape can lead to selective translation of mRNAs that encode proteins involved in drug resistance mechanisms.^10^ Consequently, understanding the nuances of rRNA modifications in cancer cells opens new avenues for therapeutic interventions. Targeting the specific rRNA modifications or the enzymes responsible for these modifications presents a promising strategy for developing novel anticancer therapies. This approach holds the potential to restore normal protein synthesis in cancer cells, thereby inhibiting their proliferation and survival.

In this review, we provide a comprehensive and critical evaluation of rRNA modifications and their impact on cellular functions, with a particular emphasis on their aberrant deregulation in cancer. The uniqueness of our review is highlighted by several aspects. Firstly, this is the first systematic summary and analysis of rRNA modifications in the context of cancer, offering a novel perspective on how these modifications influence cancer development and progression. Secondly, we delve into the role of mitochondrial rRNA modifications in regulating cellular functions and their dysregulation in cancer. This aspect provides novel insights into the less‐explored area of mitochondrial rRNA and its significant implications in oncogenesis. Thirdly, we explore the mechanisms by which rRNA modifications regulate both the function of rRNA itself and broader cellular physiological processes. Lastly, we discuss innovative and potential therapeutic strategies targeting rRNA modifications for cancer treatment. This discussion opens new avenues for targeted therapies in oncology, harnessing the nuances of rRNA modification dynamics.

## 2 OVERVIEW OF RRNA MODIFICATION

rRNA is a key molecule in cellular protein synthesis, composed of nucleotides including sugar molecules, phosphate groups, and nitrogenous bases, and constitutes 80% of the total RNA in mammalian cells.^11^ As a crucial component of the protein synthesis apparatus in cells, rRNA works in concert with ribosomal proteins. Advancements in biotechnologies such as genomics, transcriptomics, epitranscriptomics, and proteomics have facilitated an in‐depth exploration of rRNA. Considered one of the most comprehensive and universal non‐coding RNAs, rRNA significantly influences cellular physical characteristics and physiological functions. As a nucleic acid ubiquitously present in eukaryotic cells, rRNA plays a critical role in ribosome synthesis and assembly, thereby being fundamental to protein synthesis. Eukaryotic cells contain ribosomes that consist of two parts: a small 40S subunit, which includes 18S rRNA, and a larger 60S subunit, composed of 28S, 5.8S, and 5S rRNAs, along with various proteins.^12^ In contrast, prokaryotic cells, such as bacteria, have smaller ribosomes. These are made up of a 30S small subunit, which contains 16S rRNA, and a 50S large subunit, which includes 23S and 5S rRNAs and proteins. Interestingly, mitochondria within eukaryotic cells have their own unique ribosomes, which closely resemble those found in prokaryotes.^13^ This similarity supports the Endosymbiont Theory, which proposes that mitochondria evolved from a symbiotic relationship between an ancestral eukaryotic cell and a prokaryotic organism. As a result, the ribosomes in human mitochondria are composed of a 28S small subunit, including 12S rRNA, and a 39S large subunit with 16S rRNA, mirroring the structure of prokaryotic ribosomes.^14^ Highly conserved throughout evolution in terms of overall structure and composition, these molecules not only provide structural support within the ribosome but also ensure the accurate generation of proteins during protein synthesis, maintaining normal cell function.^15^

Unlike widely studied RNA processing mechanisms such as splicing, capping, and polyadenylation, the chemical modifications of bases and ribose in RNA are significant for the structure‐function relationship of RNA.^16^ These modifications are highly regulated, imparting specificity to different types of RNA. RNA modifications, a widespread biological phenomenon present in most RNA types, currently include over 170 different types.^17^ The conserved structure of rRNA depends not only on ribosomal proteins but also, to some extent, on specific chemical modifications on particular rRNA nucleotides, including 2′‐O‐methylation (2΄‐OMe), psudouridylation, and N6‐methylation (m^6^A). These modifications contribute to the stability of modified bases in specific orientations. It has been demonstrated that approximately 2% of nucleotides in rRNA undergo various types of modifications, making it the second most modified RNA type after tRNA.^18^ These modifications primarily occur in areas critical to ribosomal function, effectively stabilising ribosomal structure and playing an essential role in translation fidelity.^19^, ^20^ rRNA modifications typically affect early steps of ribosome biogenesis but also subtly modulate the catalytic function of ribosomes in translation through specific post‐transcriptional modifications.^21^ Recent studies have summarised that these modifications predominantly occur at specific sites in rRNA molecules, with over 100 rRNA uridines known to be targets of psudouridylation. Concentrated in functional regions of the ribosome, especially crucial functional areas like the peptidyl transferase center and decoding site, these modifications confer greater conformational stability to rRNA, ensuring correct folding and interaction with ribosomal proteins.^22^ Furthermore, these modifications are not just for maintaining the basic structure of the ribosome; they dynamically participate in the regulation of gene expression. By adjusting the level of rRNA modifications, cells can alter the structure of ribosomes, affecting their activity, and play roles in translation accuracy. The primary rRNA modifications include 2′‐O‐methylation, where methyl groups are added to the 2′ hydroxyl group of ribose sugars.^23^ This modification is crucial for maintaining the RNA's proper three‐dimensional structure, which is essential for the correct assembly and stable function of the ribosome. Pseudouridylation involves the conversion of uridine into pseudouridine, altering the RNA backbone to enhance molecular stability and improve the ribosome's accuracy in translating genetic messages.^24^, ^25^, ^26^ Moreover, rRNA is subject to base modifications, which include methylation at various nucleobases—adjusting their hydrogen bonding patterns and stacking interactions essential for ribosome mechanics—as well as acetylation (acN) and Aminocarboxypropylation (acpN).^27^ These modifications alter the electrochemical characteristics of the rRNA, affecting its interaction with translational machinery and enabling fine‐tuned responses to physiological demands.^28^, ^29^

These modifications are strategically located in functionally crucial regions of the rRNA, particularly the peptidyl transferase center and the decoding site.^30^ Such placement ensures that these modifications contribute maximally to ribosomal performance, facilitating high fidelity in protein synthesis.^31^ By regulating these chemical modifications, cells can dynamically modulate ribosome function in response to environmental stimuli and developmental cues, thereby controlling gene expression at the translational level. This dynamic regulation underscores the critical role of rRNA modifications in adapting cellular machinery to meet metabolic and environmental challenges, highlighting their profound impact on cellular health and disease states.^32^

## 3 TYPES OF RRNA MODIFICATION AND DETECTION TECHNIQUES

**FIGURE 1:**
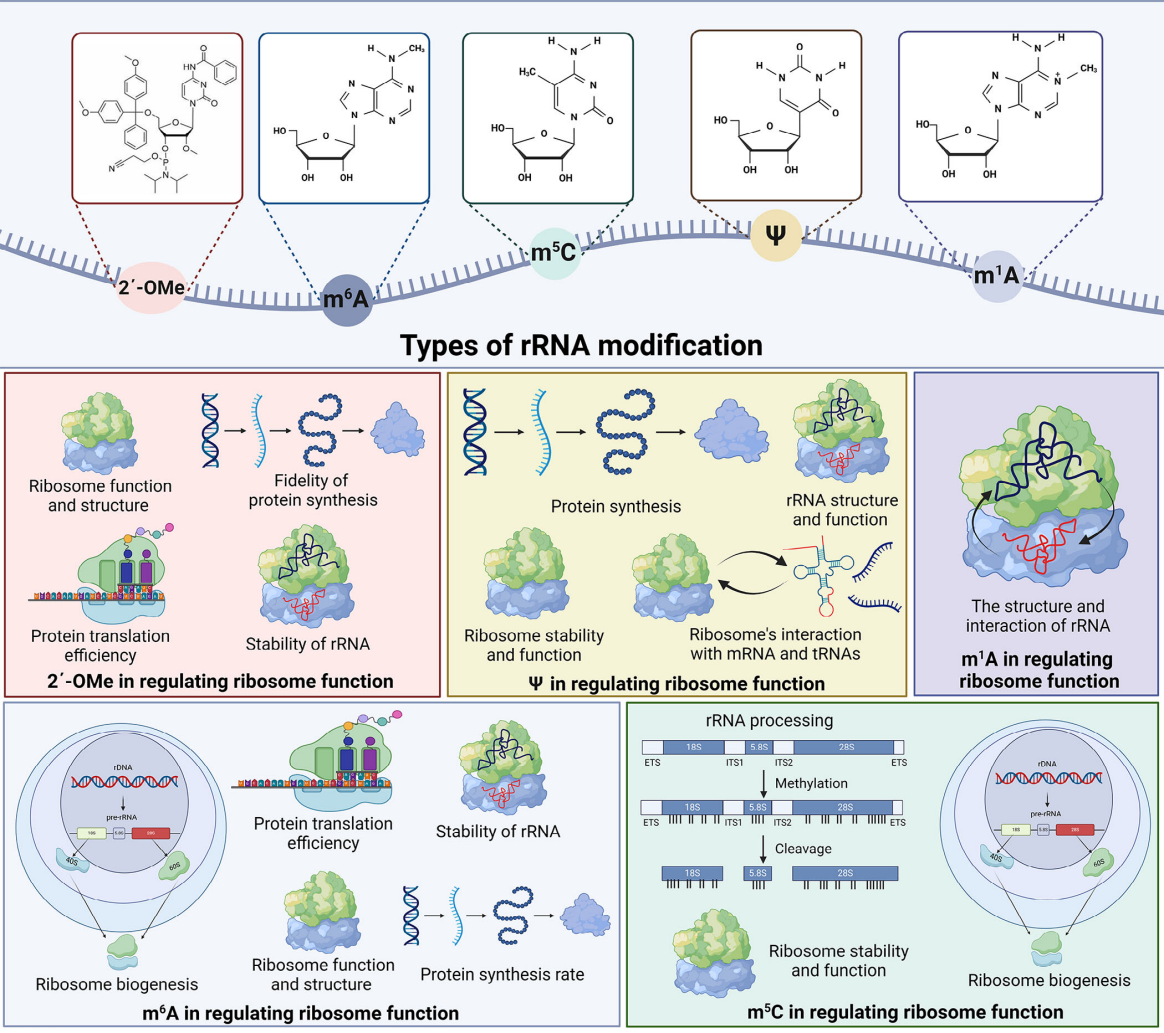
Type of rRNA modifications and their impact on ribosome function. rRNA undergoes various modifications, notably 2′‐OMe, m^6^A, m^5^C, Ψ, and m^1^A, which are pivotal in regulating ribosome functionality and cellular processes. These modifications are essential in maintaining rRNA stability, facilitating rRNA processing, and enhancing the fidelity and efficiency of protein synthesis. Furthermore, they also contribute significantly to ribosome biogenesis, and playing a crucial role in maintaining the structural integrity and functionality of ribosomes. Created with BioRender.com.

rRNA modifications, such as methylation and pseudouridylation, play crucial roles in regulating ribosome function (Figure 1). These modifications can impact the accuracy and efficiency of protein synthesis by altering the structure and dynamics of the ribosome. Furthermore, variations in rRNA modifications are often linked to changes in cellular response under different physiological conditions, reflecting their importance in maintaining cellular homeostasis.

### 3.1 rRNA 2΄‐OMe modification

In the realm of rRNA modifications, 2΄‐OMe stands out as a critical biochemical alteration that plays a vital role in the functional integrity and efficiency of the ribosome. This modification, characterised by the addition of a methyl group to the 2′‐hydroxyl group of the ribose sugar in the backbone of rRNA, is facilitated by a complex interplay of enzymes, including methyltransferases guided by small nucleolar RNAs (snoRNAs).^33^ The 2΄‐OMe of rRNA is not randomly distributed but occurs at specific nucleotide positions, often at functionally crucial sites of the ribosome.^34^ This modification enhances the stability of rRNA, contributes to the maintenance of the correct structure of the ribosome, and ensures the fidelity of protein synthesis.^35^ It plays a significant role in fine‐tuning the ribosome's response to various cellular conditions and stressors, thereby influencing overall protein translation efficiency.^32^ For instance, altering 2΄‐OMe patterns in rRNA affects ribosome function and fidelity, influencing various ribosomal conformational states. Interestingly, differential 2΄‐OMe of rRNA, identified by RiboMeth‐seq, is a key source of ribosome heterogeneity in human cells. This methylation varies in response to factors like MYC oncogene expression and affects the translation of specific mRNAs and cellular phenotypes. Thus, variations in rRNA 2΄‐OMe lead to functionally specialised ribosomes, highlighting the role of rRNA modifications in precisely regulating translation.^36^ Similarly, in an analysis of over 15 human cell lines, 2΄‐OMe in rRNA is classified into four variability groups, suggesting ‘specialised ribosomes’. About 1/3 of these modifications are consistently stable across all samples, while the rest show varying degrees of change. Stable modifications predominantly occur in crucial ribosomal functional sites, whereas more variable ones are on the periphery, indicating a potential link between rRNA modification patterns and specific ribosome functions.^37^

DBP3, a DEAD box helicase, is essential for processing snoRNAs and 2΄‐OMe in 25S rRNA. In its absence, snoRNP accumulation on pre‐ribosomes interferes with rRNA modifications. snoRNAs for DBP3‐dependent modifications are primarily linked to pre‐ribosomes, with DBP3 deficiency impeding rRNA methylation by affecting snoRNP recycling and target site access. This reflects a stochastic snoRNP interaction with pre‐rRNA, crucial for early ribosome assembly.^38^ In addition, GPATCH4 regulates the DEAH box helicase DHX15, affecting 2΄‐OMe in rRNA and snRNA. Loss of GPATCH4 disrupts methylation, impacting ribosome function and pre‐mRNA splicing, with some effects dependent on DHX15's ATPase activity.^39^ Moreover, EZH2, known as a transcription repressor and lysine methyltransferase, interacts with fibrillarin (FBL) to regulate translation by enhancing rRNA 2΄‐OMe. This interaction also strengthens the FBL‐NOP56 interaction, crucial for small nucleolar ribonucleoprotein (snoRNP) assembly. EZH2 deficiency in cancer cells impairs global translation and internal ribosome entry site (IRES)‐dependent initiation, revealing its novel role in translational regulation through rRNA modification.^40^ Importantly, methylation of G2922 in rRNA is crucial for ribosome assembly, particularly influencing the large subunit's assembly and nuclear export. Interactions between nuclear GTPase NOG2 with the 2΄‐OMe base Gm2922 are vital, with certain amino acid changes in NOG2 able to bypass the need for this methylation.^41^ This highlights the importance of a single RNA modification as a key regulatory checkpoint in ribosome biogenesis. In addition, the methyltransferase SPB1 modifies G2922 in the rRNA A‐loop, a crucial step in 60S ribosomal subunit biogenesis. A catalytically deficient SPB1 mutant leads to a significant 60S biogenesis defect due to unmethylated G2922 triggering premature activation of NOG2 GTPase activity. This premature activation hinders Nog2's efficient binding to early nucleoplasmic 60S intermediates.^42^

### 3.2 rRNA m^6^A modification

The m^6^A modification in rRNA is a distinctive post‐transcriptional modification influencing various cellular processes. m^6^A modification, occurring at adenosine residues, plays a crucial role in modulating the structure and function of ribosomes. It contributes to the fine‐tuning of translational efficiency and fidelity, impacting protein synthesis rates under different cellular conditions.^43^

METTL3, modifying adenosine 196 in the 47S rRNA precursor, accelerates pre‐rRNA processing rates while maintaining stable levels of rRNA processing components and mature rRNA. This leads to reduced 47S and 45S pre‐rRNAs and increased mature rRNA in cells, indicating its key role in the early stages of ribosome biogenesis and the quality control of pre‐rRNA processing.^44^ Additionally, ZCCHC4 is identified as responsible for the m^6^A modification in human 28S rRNA at position A4220. This modification, crucial for RNA structure and function, is shown to have significant effects on mRNA translation. The absence of this specific m^6^A modification in 28S rRNA leads to altered codon‐specific translation dynamics and shifts in gene expression, emphasising the vital role of m^6^A in regulating the translation of mRNA.^45^ Interestingly, quantitative mass spectrometry has uncovered mono‐methylation alongside the typical di‐methylation (m^6^_2_A) at the 3′ end of 18S rRNA. This m^6^A modification notably increases under sulphur starvation in yeast and mammalian cells. Ribosomes with m^6^A exhibit distinct translation patterns from those with m^6^_2_A, particularly favouring sulphur metabolism genes, suggesting methylation variability as a mechanism regulating translation.^46^

### 3.3 rRNA m^5^C modification

The m^5^C modification in rRNA is a key epigenetic marker that affects ribosome biogenesis and function. This modification, catalysed by specific methyltransferases, predominantly occurs in the more evolutionarily conserved regions of rRNA, suggesting a fundamental role in ribosome stability and interaction with tRNA during protein synthesis.^47^ For instance, NOP2/NSUN1 catalyses m^5^C addition at position 4447 on 28S rRNA and aids pre‐rRNA processing by recruiting U3 and U8 snoRNAs into snoRNP complexes. Interestingly, both active and inactive NOP2/NSUN1 can rectify rRNA processing defects, suggesting that its catalytic activity is not crucial for ribosome synthesis. This underscores NOP2/NSUN1's diverse roles in rRNA modification and ribosome assembly.^48^ Similarly, human p120 and yeast NOP2, members of the NOP2/NSUN/NOL1 protein family, exhibit m^5^C‐methyltransferase activity, crucial for m^5^C formation in 25S rRNA. NOP2p and RCM1p are confirmed to catalyse m^5^C formation in rRNA, with NOP2's presence being vital for pre‐rRNA processing. This highlights NOP2's dual role in ribosome synthesis and the importance of m^5^C modifications in ribosome quality control.^49^ Interestingly, YTHDF2 has been identified as a key protein that recognises m^5^C in RNA, especially in rRNA. YTHDF2 binds to m^5^C with lower affinity compared to its binding to m^6^A. Knocking out YTHDF2 leads to increased m^5^C methylation in rRNA, underscoring its role in rRNA processing and in regulating the function of m^5^C in RNA biology.^50^

### 3.4 rRNA Ψ modifications

Ψ, the isomer of uridine, is one of the most prevalent modifications in rRNA, playing a pivotal role in ribosome structure and function. The conversion of specific uridine residues to Ψ, catalysed by pseudouridine synthases, leads to enhanced hydrogen bonding and ribosomal stability.^51^ This modification is critical for the proper folding and three‐dimensional structure of rRNA, thereby influencing the ribosome's interaction with mRNA and tRNAs.^52^, ^53^ Hypopseudouridylated ribosomes, analysed through cryo‐electron microscopy, demonstrate that the lack of pseudouridine in rRNA leads to the loss of crucial molecular interactions. This results in an unfavourable conformation for decoding, especially during translocase and elongation factor activities. The presence of pseudouridine at the P‐site uridine partially mitigates these effects, underscoring pseudouridine's vital role in rRNA function and broader RNA processes.^54^ In trypanosomes, genome‐wide studies show that pseudouridine modifications on rRNA, particularly on helix 69, are crucial for survival and affect ribosome composition. Knockouts of snoRNAs guiding Ψ modifications alter the translation of specific proteins. This suggests that rRNA modifications can create ribosomes tailored for translating certain proteins, emphasising the role of psudouridylation in ribosomal function and selective protein synthesis.^55^

### 3.5 Other rRNA modifications

In addition to the typical rRNA modifications like 2΄‐OMe, Ψ, and m^5^C, there are several other less common but equally crucial rRNA modifications that play important roles in ribosome function and cellular processes. These include base acetylation, a modification that influences the hydrogen bonding patterns of nucleotides, and base methylation at positions other than the 5th carbon, such as 1‐methyladenosine (m^1^A) and 3‐methylcytidine (m^3^C), which can affect the structure and interactions of rRNA.^56^, ^57^ Additionally, there are complex modifications like N6‐threonylcarbamoyladenosine, which occur in tRNA but can also be seen in rRNA, contributing to the stability and accuracy of protein synthesis.^58^ For instance, SNORD13 is essential for acetylating a specific cytidine in 18S rRNA, a process mediated by NAT10, but this modification is not vital for human cell growth, ribosome biogenesis, or translation. Cross‐species analysis reveals diverse SNORD13 genes, including an unusual variant in *Drosophila* and absent rRNA acetylation in *Caenorhabditis elegans*, highlighting the variable evolutionary significance of this rRNA modification.^59^ In addition, the m^1^A modification in rRNA, catalysed by RRP8 in yeast and nucleomethylin in humans, is vital for ribosome structure and function. This modification affects the conformation of yeast 25S rRNA, influencing translation initiation and the production of key proteins.^60^

### 3.6 Methods for detecting rRNA modifications

Next‐generation sequencing (NGS) and mass spectrometry, renowned for their precision and comprehensive analysis, are commonly used for identifying rRNA modifications. The strength of NGS lies in its high throughput capabilities and shorter read lengths, which are particularly suitable for detecting a wide array of rRNA modifications.^61^ There are typically three NGS‐based approaches used for detecting rRNA modifications. The first method detects naturally occurring modified nucleotides through reverse transcription signatures, revealing changes in modified nucleotides readout caused by RNA‐dependent RNA polymerase during primer extension, exemplified by the conversion of adenosine to inosine. The second approach, including techniques like bisulfite‐induced deletion sequencing (BID‐seq), detects rRNA modifications through chemically induced reverse transcription signatures or specific RNA ribose‐phosphate backbone cleavage.^62^ BID‐seq quantitatively converts specific RNA modifications, such as psudouridylation, into detectable adducts. This conversion causes base deletion signals at Ψ sites during reverse transcription, enabling quantitative sequencing of Ψ modifications on RNA with single‐nucleotide resolution.^63^ The third method involves classical affinity/immunoprecipitation‐based protocols, employing antibodies or enzymes recognising RNA modifications. For example, methylated RNA immunoprecipitation sequencing (MeRIP‐Seq) enriches methylated fragments via immunoprecipitation, combined with high‐throughput sequencing for studying regions of mRNA with m^6^A methylation modifications.^64^, ^65^ Notably, the development of next‐next generation sequencing (NNGS) promises advancements in RNA modification detection with longer read lengths and higher resolution than NGS.^66^

Liquid chromatography‐tandem mass spectrometry (LC‐MS/MS) is a crucial tool for rRNA modification analysis, offering comprehensive sample analysis. This process includes extracting and purifying rRNA from samples, followed by degradation into nucleosides or oligonucleotides. These fragments undergo liquid chromatography to separate modified from unmodified nucleosides. Subsequently, in a mass spectrometer, these components are ionised and analysed based on their mass‐to‐charge ratio. The sensitivity and precision of LC‐MS/MS make it ideal for identifying and quantifying rRNA modifications.^67^ To enhance the reliability of experimental outcomes, stable isotope‐labelled nucleosides are often employed as internal standards, offsetting potential biases that may arise during sample processing and analysis. Interestingly, LC‐MS/MS has been used to explore the mechanism by which cancer stem cells adapt to harsh environments through RNA modification, identifying specific RNA markers associated with CSC enrichment.^68^

In addition to these conventional methods, innovative techniques for rRNA modification detection and quantification have been developed. A recent example is a new quantitative reverse transcription PCR‐based method, enabling sensitive assessment of ribose 2′‐OMe levels at specific rRNA sites with single‐nucleotide resolution. This technique requires minimal RNA amounts, offering a simpler, more affordable approach for rRNA modification profiling.^69^ Another novel method, HydraPsiSeq, facilitates precise and specific Ψ mapping in RNA. With its low RNA requirement, it is well‐suited for high‐throughput studies and has identified significant Ψ variations in human rRNAs. Moreover, the Rosetta stepwise Monte Carlo method, a computational rRNA design technique, is critical in rRNA engineering. It efficiently screens mutant ribosomes, especially those targeting the peptidyl transferase center, marking a significant leap in understanding ribosome modifications and their applications in synthetic biology and cellular engineering.^70^ Furthermore, the comparison of modified and pseudouridine‐free *Saccharomyces cerevisiae* ribosomes using electron cryomicroscopy reveals that pseudouridine plays a crucial role in stabilising rRNA structures, which is essential for their proper function. The absence of pseudouridine resulted in abnormal ribosome movements and translation deficiencies, underscoring its critical role in ribosome activity.^71^

## 4 THE ROLE OF RRNA MODIFICATION IN REGULATING BIOLOGICAL AND CELLULAR FUNCTION

**FIGURE 2:**
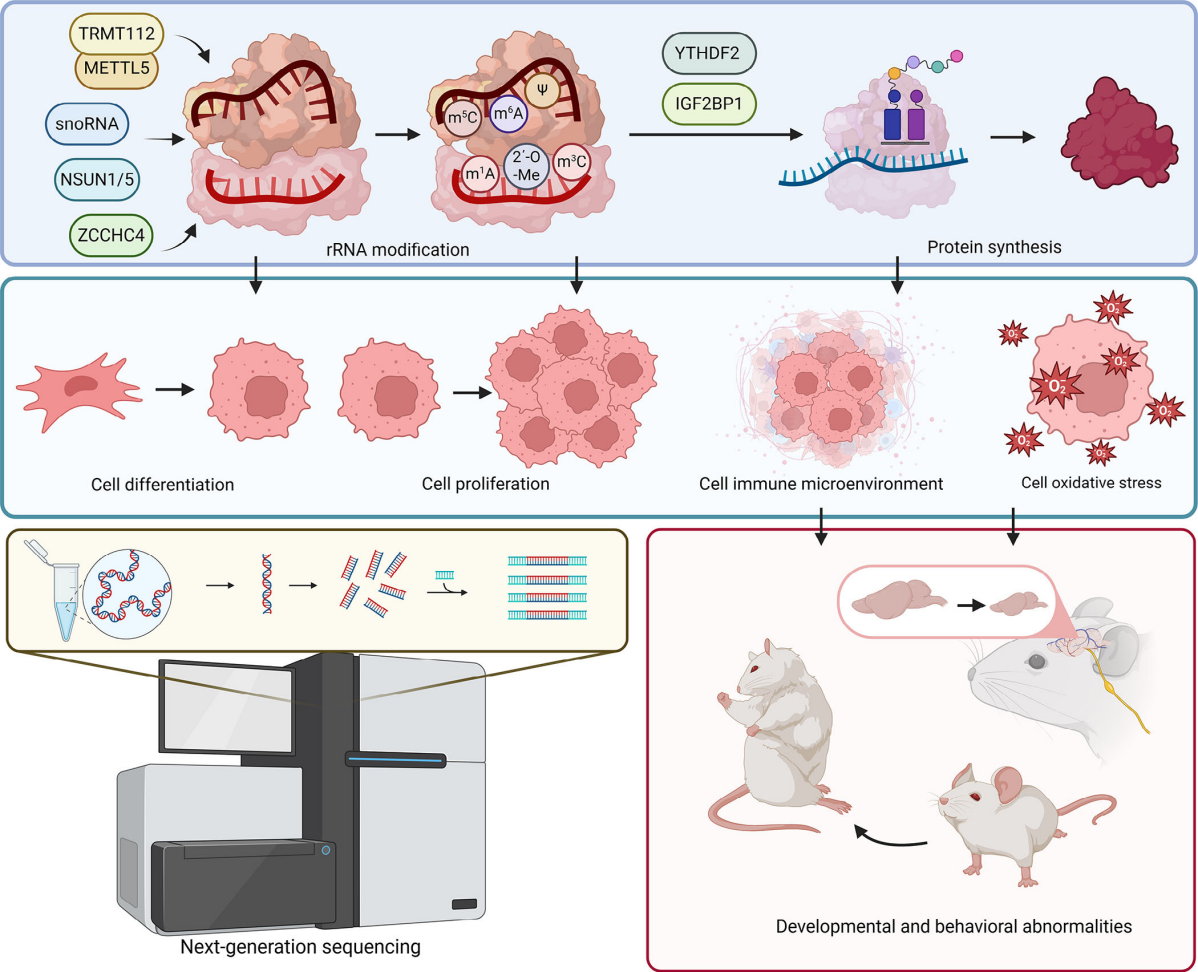
Regulatory role of rRNA modifications in biological and cellular functions. Various modifications of rRNA are known to play critical roles in the regulation of cellular functions. Through next‐generation sequencing, it has been elucidated that numerous RNA modification enzymes, such as TRMT112, METTL5, NSUN1/5, ZCCHC4 etc., along with snoRNAs, play pivotal roles in mediating rRNA modification. This dynamic modification process intricately regulates the synthesis of related proteins, thereby influencing diverse cellular functions including cell proliferation, differentiation, immune response, and oxidative stress response. Consequently, these modifications exert profound effects on an individual's developmental processes, behavioural patterns, and overall survival and growth status. Created with BioRender.com.

rRNA modifications play a critical role in the regulation of cellular functions, significantly impacting the efficiency and fidelity of protein synthesis. Notably, the diversity and distribution of these modifications are not static but dynamically regulated in response to cellular needs, developmental cues, or environmental stresses. This dynamic regulation allows cells to modulate protein synthesis rates and adapt to changes, influencing key biological processes such as cell differentiation, development, growth, and senescence (Figure 2). During mouse brain development and human embryonic stem cell differentiation, variations in rRNA 2΄‐OMe highlight ribosome heterogeneity's role in translational regulation. Specific rRNA methylation changes impact neuronal differentiation by affecting fragile X mental retardation protein (FMRP) association with ribosomes and altering WNT pathway mRNA translation. This highlights the significance of rRNA 2′‐O‐Me in directing translation and cell fate during early development.^72^ Similarly, NSUN‐1, identified as the enzyme modifying 26S rRNA at position C2982 in *Caenorhabditis elegans*, impacts organismal physiology. Its depletion, while improving thermotolerance and extending lifespan, leads to reduced body size and fecundity, and specific changes in mRNA translation. This includes decreased collagen production and cuticle integrity, underscoring the profound effects of rRNA methylation on development and physiology.^73^ Additionally, RiboMeth‐seq profiling of human dermal fibroblasts reveals distinct 2′‐O‐Me patterns of rRNA associated with cellular senescence and quiescence. Alterations in rRNA methylation, guided by snoRNAs like SNORD87 and SNORD88 variants, significantly affect cell proliferation, indicating a critical role of rRNA modifications in regulating primary cell growth phenotypes.^74^ Interestingly, GPATCH4, identified as a stimulatory cofactor of the DHX15 RNA helicase, plays a key role in 2′‐O‐Me of rRNA and snRNA, regulating protein synthesis and cell growth.^75^ Notably, environmental stress can also significantly impact rRNA modifications. For instance, hypoxia induces changes in rRNA methylation patterns, promoting the formation of “specialised ribosomes” with unique functions. These differentially methylated ribosomes enhance IRES‐mediated translation of key genes, like VEGF‐C, which are crucial for tumour progression.^76^

METTL5 is identified as catalysing m^6^A in 18S rRNA at position A1832. Its absence in mouse embryonic stem cells leads to reduced global translation, loss of pluripotency, and impaired differentiation. METTL5‐deficient mice show developmental and behavioural abnormalities, mirroring symptoms in patients with METTL5 DNA variants.^77^ In addition, METTL5‐mediated m^6^A modification on 18S rRNA regulates the translation of FBXW7, with METTL5 deficiency leading to reduced FBXW7 levels and delayed the onset of cell differentiation onset.^78^ Moreover, in METTL5 knockout mouse models, defects in craniofacial and nervous development are observed, along with intellectual disabilities and disrupted myelination processes.^79^ Furthermore, mutations in METTL5, linked to microcephaly and intellectual disability, disrupt its interaction with tRNA methyltransferase activator subunit 11−2 (TRMT112), affecting ribosomal function.^80^ Loss of METTL5 influences gene expression at the translational level in human cancer cell lines and mice, with METTL5 knockout mice exhibiting reduced body size and metabolic defects.^81^ These findings establish METTL5's crucial role in m^6^A modification of rRNA, affecting stemness, differentiation, and disease development.

Recently, RiboMeth‐seq profiling of human dermal fibroblasts reveals distinct 2′‐O‐Me patterns of rRNA associated with cellular senescence and quiescence. Alterations in rRNA methylation, guided by snoRNAs like SNORD87 and SNORD88 variants, significantly affect cell proliferation, indicating a critical role of rRNA modifications in regulating primary cell growth phenotypes.^74^ Interestingly, GPATCH4, identified as a stimulatory cofactor of the DHX15 RNA helicase, plays a key role in 2′‐O‐Me of rRNA and snRNA, regulating protein synthesis and cell growth. This regulation involves both DHX15‐dependent and independent pathways, with DHX15's ATPase activity necessary for efficient methylation.^75^

## 5 MITOCHONDRIAL RRNA MODIFICATION

**FIGURE 3:**
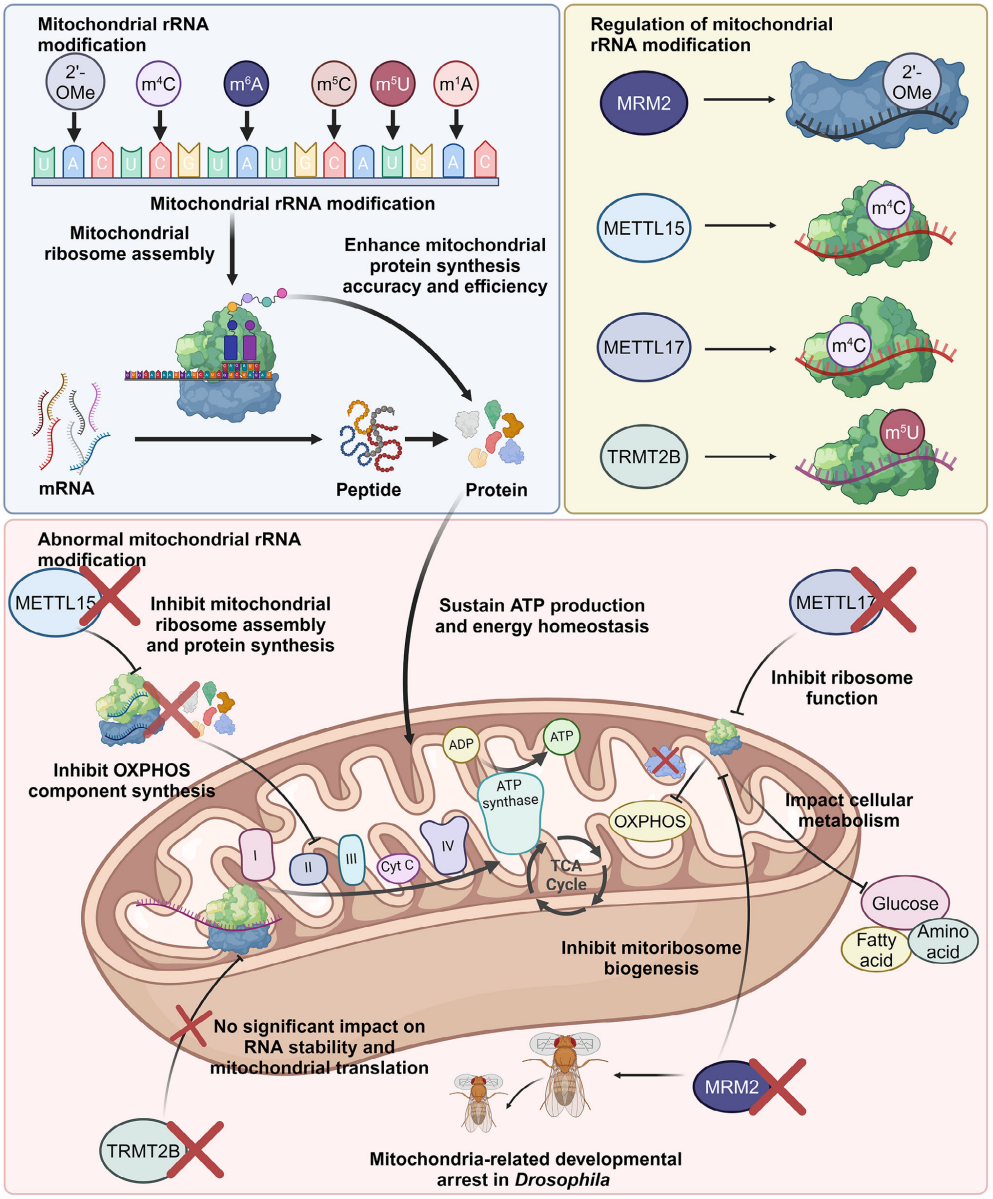
Regulation of mitochondrial rRNA modification. Mitochondrial rRNA undergoes various modifications, including 2′‐OMe, m^4^C, m^6^A, m^5^C, m^5^U, m^1^A, among others. Proper modifications of mitochondrial rRNA are crucial for the biogenesis of mitochondrial ribosomes, enhancing protein synthesis efficiency and facilitating the formation and function of key components in the mitochondrial respiratory chain. Alterations in the expression levels of enzymes such as METTL15, METTL17, and MRM2 lead to changes in mt‐rRNA modification, impacting ribosome assembly and protein translation. This, in turn, can impair essential mitochondrial functions, notably OXPHOS. TRMT2B, which catalyses the m^5^U modification of human 12S mitochondrial rRNA, plays a regulatory role rather than being essential for mitochondrial rRNA stability and mitochondrial translation. Created with BioRender.com.

Mitochondrial rRNA modifications play a critical role in cellular energy metabolism by optimising mitochondrial ribosome function (Figure 3).^82^ These modifications enhance the accuracy and efficiency of mitochondrial protein synthesis, essential for the respiratory chain's assembly and function.^83^ Consequently, they directly impact ATP production and cellular energy homeostasis. Disruptions in these modifications can lead to mitochondrial dysfunction, contributing to various metabolic and neurodegenerative diseases.^84^ Thus, mitochondrial rRNA modifications are crucial for maintaining cellular energy balance and mitochondrial health.

METTL15, responsible for cytosine‐N4 methylation (m^4^C) at position 839 in 12S mitochondrial rRNA, is essential for mitochondrial ribosome assembly and protein synthesis.^85^, ^86^ Evolutionary analysis shows divergent substrate preferences between METTL15 and its bacterial ortholog, rsmH. While the loss of m^4^C methylation has a modest impact on bacterial ribosomes, METTL15 depletion impairs mitochondrial mRNA translation and reduces respiratory capacity, highlighting distinct roles for m^4^C methylation in prokaryotic and eukaryotic mitochondrial ribosomes.^87^ Additionally, METTL17 interacts with 12S mitochondrial ribosomal RNA and mitochondrial ribosome small subunits, maintaining their stability. METTL17's loss reduces specific methylation in 12S mt‐rRNA, impairing mitochondrial ribosome function and translation of mitochondrial proteins. This leads to altered mitochondrial oxidative phosphorylation and cellular metabolism, crucial for maintaining pluripotency in embryonic stem cells.^88^ Moreover, 2΄‐OMe in human mitochondrial 16S mt‐rRNA, essential for mitochondrial respiration and mitoribosome biogenesis, is introduced by MRM1, MRM2, and MRM3. MRM2, in particular, is vital for mtLSU assembly, as its absence leads to disordered RNA domains and developmental arrest in *Drosophila*.^89^ Notably, TRMT2B, a nuclear‐encoded enzyme, catalyses 5‐methyluridine (m^5^U) modifications in human mitochondrial tRNAs and 12S rRNA. However, its knockout in human cells shows no significant impact on RNA stability, mitochondrial translation, or cellular growth, indicating a regulatory but non‐critical role of m^5^U in mitochondrial rRNA function.^90^

## 6 ABERRANT RRNA MODIFICATION AS A CANCER FEATURE

### 6.1 Aberrant global rRNA modification profile in cancer

**FIGURE 4:**
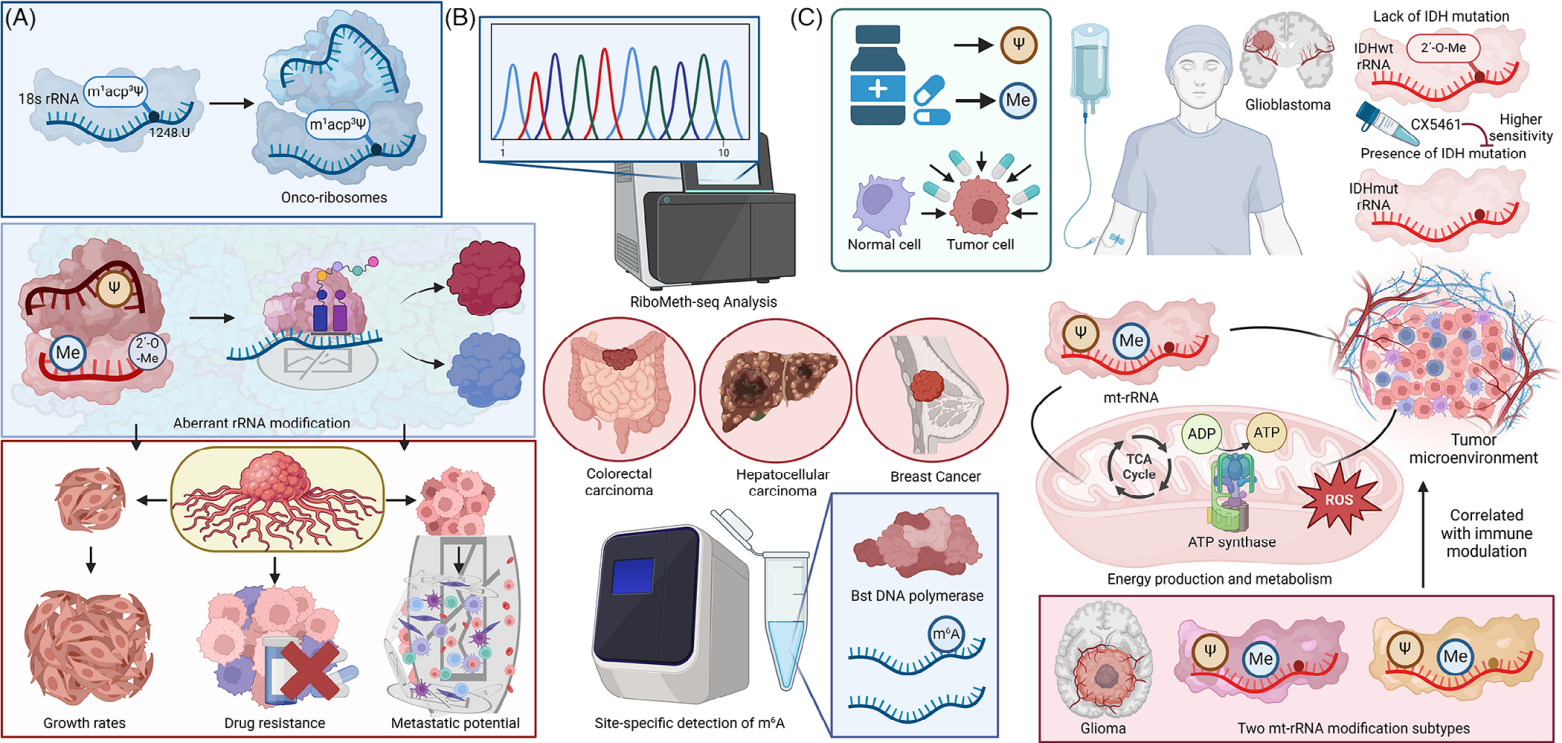
Clinical relevance of rRNA modifications in cancer. rRNA modifications play a pivotal role in regulating protein synthesis and cellular functionality, making significant contributions to the development and progression of tumours. A. Increasingly, these modifications are recognised as hallmarks of cancer, influencing drug resistance, tumour development, and metastasis. Aberrant rRNA modifications, such as single nucleotide variants in 18S rRNA impacting the ancient m^1^acp^3^Ψ modification in colorectal cancer, is a notable example. B. Advanced techniques like and RiboMeth‐seq has revealed variability in Ψ and 2′‐O‐methylation levels in cancer. C. Specific rRNA modifications in HGG (high‐grade glioma) emerge as promising therapeutic targets. There is exploration into the potential of using drugs to modulate enzyme activities responsible for rRNA modifications, utilising rRNA modification patterns as predictive biomarkers for treatment efficacy. Notably, in glioma, the profound impact of mitochondrial rRNA modifications on mitochondrial functionality significantly influences energy production, metabolism, and the tumour microenvironment. Created with BioRender.com.

Aberrant rRNA modification is increasingly recognised as a hallmark of cancer, playing a critical role in tumourigenesis and progression (Table 1). In cancer cells, alterations in the patterns of rRNA modifications, such as methylation and psudouridylation, disrupt normal ribosome function, leading to changes in protein synthesis that favour oncogenic processes (Figure 4). Using RBS‐Seq, variability in Ψ and 2΄‐OMe levels of rRNA is identified in breast cancer samples. Hypermodification at specific rRNA sites correlates with certain tumour characteristics, indicating a potential role in cancer progression. The most stable and conserved pseudouridine sites are particularly significant, underscoring the importance of these specific rRNA modifications in understanding ribosomal changes in tumours.^91^ Similarly, RiboMeth‐seq analysis reveals significant hypomethylation at numerous 2′‐O‐Me sites in ribosomal RNA from diffuse large B‐cell lymphoma (DLBCL) cell lines, particularly in the ABC subtype compared to the GBC subtype. Such ribosomal RNA modification patterns, also confirmed in patient tumour samples, suggest cell growth and tumour‐specific alterations.^92^

**TABLE 1:** The clinical significance of rRNA modifications in cancer.

Cancer type	rRNA type	Nucleotide	Modification type	Clinical significance	Ref
CRC	18S rRNA	1248.U	Ψ	A chemotherapeutic target for treating cancer	124
Breast cancer	18S rRNA	Ψ34–218	Ψ	Distinguishes different patient clusters	91
Breast cancer	28S rRNA	Ψ4598–4975	Ψ	Distinguishes different patient clusters	91
Breast cancer	18S rRNA	Am576	2΄‐OMe	Influences tumour heterogeneity and subdivide cancer grading	95
HGG	18S rRNA	Gm1447	2΄‐OMe	Influences tumour heterogeneity	123
DLBCL	rRNA	SSU‐C1440	2′‐O‐Me	Potential drug targets for DLBCL	92

### 6.2 rRNA modification and tumour heterogeneity

rRNA modification plays a significant role in contributing to tumour heterogeneity, a key characteristic of many cancers that complicates treatment and prognosis. Tumour heterogeneity refers to the presence of diverse subpopulations of cancer cells within the same tumour, each with distinct genetic, phenotypic, and behavioural traits.^93^ Variations in rRNA modifications across different cancer cell subpopulations can lead to differences in ribosome function and, consequently, protein synthesis profiles. This diversity in protein expression patterns results in varying cellular behaviours, such as differential growth rates, metastatic potential, and drug resistance. For instance, in breast cancer, cells with high aggressivity exhibit distinct ribosomal biogenesis and function compared to their less aggressive counterparts. These highly aggressive cells show increased 45S pre‐rRNA synthesis, utilise an alternative pathway involving a 43S precursor, and demonstrate enhanced methylation at specific sites on 28S rRNA, thereby linking rRNA modifications to tumour aggressivity and heterogeneity.^94^ Importantly, the RiboMeth‐seq analysis of 195 human breast tumours reveals stable and variable 2΄‐OMe sites in rRNA, indicating ribosome variability in cancer. These methylation patterns, some tolerating absence of modification, differ within and between patients, correlating with cancer subtypes and tumour grades. This suggests that rRNA 2΄‐OMe contributes significantly to tumour heterogeneity and may offer a new molecular target in cancer treatment.^95^

### 6.3 Mitochondiral rRNA modification in cancer

In cancer, changes in mitochondrial rRNA modifications can significantly impact mitochondrial function, affecting key processes such as energy production and metabolism, crucial for the growth and spread of cancer cells.^96^, ^97^ Moreover, these modifications may influence the generation of reactive oxygen species and modulate cellular stress responses, contributing to the resilience of cancer cells in challenging environments. By gaining insights into these mitochondrial‐specific rRNA changes, it becomes possible to enhance the accuracy of cancer diagnosis and prognosis. Additionally, targeting these modifications could open new avenues for cancer therapy, thereby refining the effectiveness and precision of treatment strategies. In glioma, two mt‐rRNA modification subtypes are significantly correlated with clinicopathological characteristics, immune modulation, and prognosis. Importantly, one of these subtypes is linked with poorer survival outcomes and is characterised by a higher tumour mutational burden. This finding underscores the significance of mt‐rRNA modifications in shaping the tumour microenvironment and regulating immunotherapy response.^98^

## 7 CRITICAL REGULATORS INVOLVED IN MEDIATING RRNA MODIFICATIONS IN CANCER PROGRESSION

### 7.1 METTL5/TRMT112 complex‐mediated rRNA modification in cancer

**FIGURE 5:**
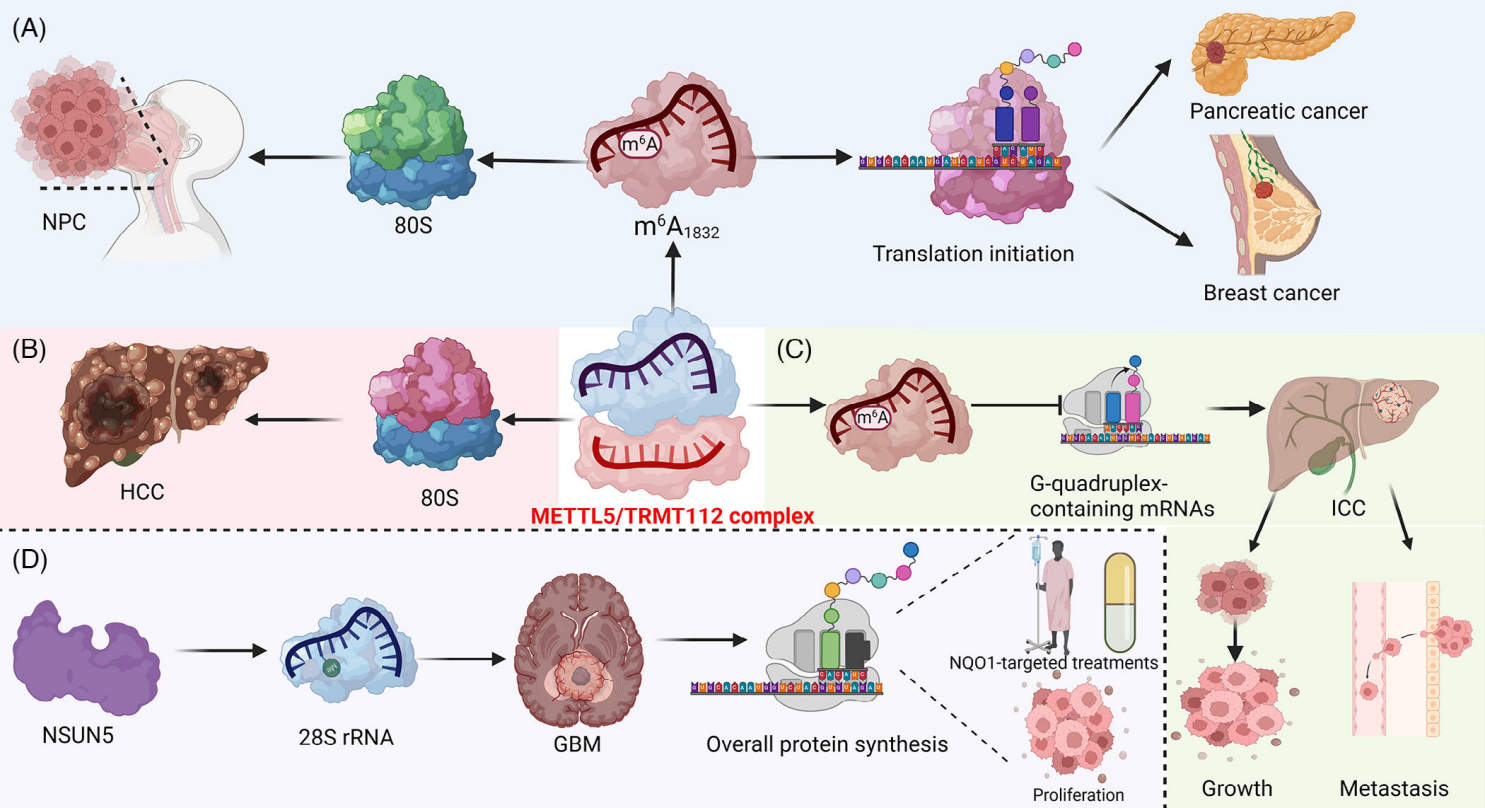
Role of METTL5/TRMT112 complex and NSUN5 in rRNA modification and cancer progression. A. METTL5‐mediated m6A modification of 18S rRNA at adenosine 1832 plays a vital role in mRNA binding, ribosome functionality, and the proliferation of cancer cells, notably in breast and pancreatic cancers. B. Furthermore, increased activity of the METTL5‐TRMT112 complex is linked to adverse cancer prognosis, mediated by compromised ribosome function and altered fatty acid metabolism. This connection suggests a promising therapeutic target for hepatocellular carcinoma. C. In intrahepatic cholangiocarcinoma, aberrant METTL5‐mediated m6A modification of 18S rRNA disrupts TGF‐β pathway translation, leading to poorer survival outcomes and accelerated cancer progression. D. Additionally, NSUN5 modifies cytosine 3782 in 28S rRNA, which enhances protein synthesis. Its overexpression is associated with accelerated growth and treatment resistance in glioma, whereas its inactivation appears to suppress tumour development and increase cellular sensitivity to therapeutic interventions. Created with BioRender.com.

The METTL5/TRMT112 complex is crucial in cancer progression, primarily through its role in rRNA modification. It specifically methylates adenine in 18S rRNA, impacting the assembly of the 80S ribosome and enhancing protein synthesis. This activity is vital for the rapid cell growth seen in cancers, positioning the complex as a significant target in cancer therapy (Figure 5) (Table 2). For instance, METTL5‐mediated modification of 18S rRNA at adenosine 1832 (m^6^A_1832_) enhances mRNA binding and ribosomal conformation, thus facilitating translation initiation and p70‐S6K activation, while its absence leads to reduced polysome abundance. Additionally, METTL5's high expression in breast cancer and necessity for cancer cell growth, along with its homolog's role in *Caenorhabditis elegans* translation, underscore its critical function in cancer progression and translational regulation.^99^ Importantly, aberrant upregulation of METTL5‐mediated 18S rRNA m^6^A modification in intrahepatic cholangiocarcinoma (ICC) correlates with poor survival. This modification selectively impairs the translation of G‐quadruplex‐containing mRNAs in the TGF‐β pathway, facilitating ICC cell growth and metastasis.^100^ Moreover, METTL5 is identified as an oncogene in pancreatic cancer, promoting cell proliferation, migration, invasion, and tumourigenesis. It catalyses critical 18S rRNA m^6^A_1832_, influencing c‐Myc translation via m^6^A modifications in c‐Myc mRNA. Additionally, METTL5 works with TRMT112 to enhance pancreatic cancer progression, positioning the METTL5/c Myc axis as a potential therapeutic target.^101^ Notably, the upregulation of the METTL5‐TRMT112 complex in various cancers correlates with poor prognosis. This alteration disrupts 80S ribosome assembly and decreases mRNA translation in fatty acid metabolism, contributing to hepatocellular carcinoma (HCC) progression. In addition, inhibiting METTL5 and its associated mediator, ACSL4, shows potential in counteracting HCC tumourigenesis.^102^ Similarly, METTL5/TRMT112‐mediated 18S rRNA m^6^A_1832_ modification facilitates 80S ribosome assembly and enhances mRNA translation, which is crucial for NPC progression. A key aspect of this mechanism is the upregulation of HSF4b translation by METTL5, which activates HSP90B1 transcription. HSP90B1 then binds with mutant p53 protein, inhibiting its degradation and contributing to NPC tumourigenesis and chemoresistance.^103^

**TABLE 2:** Critical regulators involved in mediating rRNA modifications in cancer progression.

Cancer type	Regulators	rRNA type	Nucleotide	Modification type	Clinical significance	Ref
Breast cancer	METTL5	18S rRNA	A1832	m^6^A	Promote breast cancer development	99
ICC	METTL5	18S rRNA	A1832	m^6^A	Facilitate ICC cell growth	100
Pancreatic cancer	METTL5	18S rRNA	A1832	m^6^A	Promote pancreatic cancer progression	101
HCC	METTL5‐TRMT112	18S rRNA	A1832	m^6^A	Promote HCC progression	102
NPC	METTL5‐TRMT112	18S rRNA	A1832	m^6^A	Promote NPC tumourigenesis and chemoresistance	103
GBM	NSUN5	28S rRNA	C3782	m^5^C	Enhance GBM proliferation and treatment resistance	105
Glioma	NSUN5	28S rRNA	C3782	m^5^C	NSUN5 epigenetic inactivation inhibit glioma, promote survival and enhance sensitivity to targeted treatments	106
Breast cancer	FBL	∖	∖	Changed methylation pattern	Promote breast cancer	118
HCC	ZCCHC4	28S rRNA	A4220	m^6^A	Promote HCC progression	120
Breast cancer	Dkc1	18S rRNA	U1445	Ψ	Sustain neoplastic phenotype in breast cancer promote more aggressive cellular phenotype	121

### 7.2 NSUN5‐mediated rRNA modification in cancer

NSUN5‐mediated rRNA modification plays a critical role in glioma progression and enhances therapeutic responses.^104^ For instance, elevated NSUN5 expression, which methylates cytosine 3782 of 28S rRNA, is associated with poor patient survival in GBM. Overexpression of NSUN5 enhances GBM cell proliferation and treatment resistance, whereas its knockdown diminishes these tumourigenic activities. This suggests NSUN5 as a potential therapeutic target in GBM.^105^ Similarly, the epigenetic silencing of NSUN5 results in tumour suppression and an unmethylated C3782 in 28S rRNA in glioma. This unmethylation leads to a reduction in overall protein synthesis and triggers an adaptive translational survival program under stress. Additionally, NSUN5 inactivation sensitises gliomas to NQO1‐targeted treatments and is a marker of long‐term survival in glioma patients, highlighting its significance in cancer's epitranscriptomic regulation.^106^

### 7.3 snoRNA‐mediated rRNA 2΄‐OMe

**FIGURE 6:**
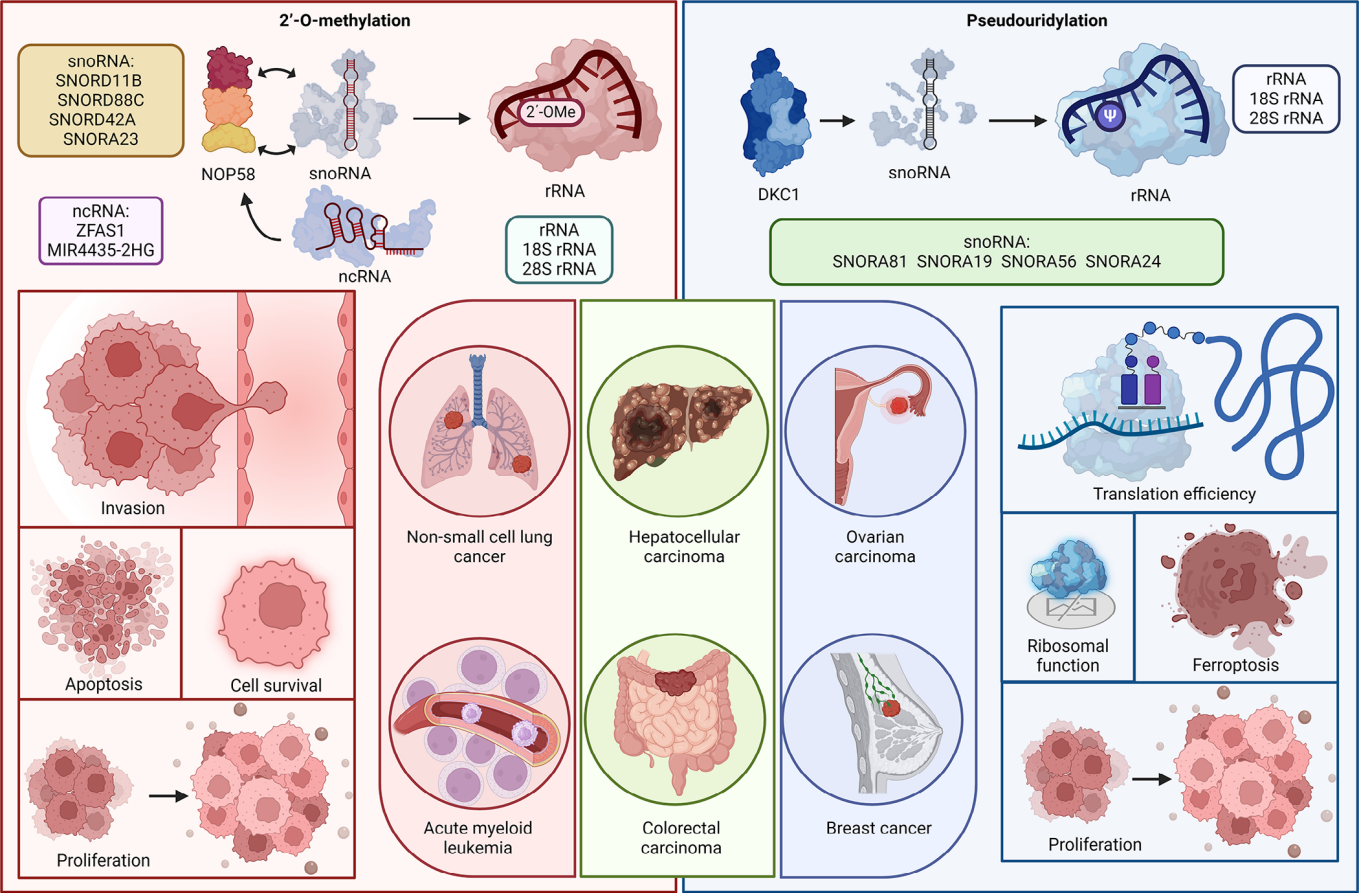
snoRNA‐mediated rRNA modification and its implications in cancer. Various snoRNAs exhibit distinct functions across different types of cancer. In rRNA modification, snoRNA promotes cancer cell proliferation and invasion while inhibiting autophagy through the enhancement of oncogene translation, among other pathways. Additionally, ncRNA indirectly regulates snoRNA by controlling the expression of NOP58. The snoRNA‐mediated rRNA 2΄‐OMe modification plays a facilitating role in cancer by promoting cell proliferation, invasion, and metastasis, potentially impacting tumour prognosis and treatment response. In addition, snoRNA‐mediated rRNA Ψ modification fosters cancer development by influencing mitochondrial function, triggering ferroptosis, and other pathways that contribute to cancer cell viability. Created with BioRender.com.

Increasing evidence demonstrates that snoRNA‐mediated rRNA 2΄‐OMe plays a critical role in cancer progression (Figure 6) (Table 3). SNORD11B is notably upregulated in CRC tissues and promotes cancer cell proliferation and invasion while reducing apoptosis. Mechanistically, SNORD11B facilitates 18S rRNA processing by mediating 2΄‐OMe modification at G509. Additionally, SNORD11B also modifies MIRLET7A1HG at the G225 site, leading to decreased tumour suppressor let‐7a‐5p expression and increased oncogene translation.^107^ Similarly, SNORD88C, upregulated in both tissue and plasma, serves as a potential non‐invasive biomarker for non‐small cell lung cancer (NSCLC). It promotes NSCLC proliferation and metastasis by enhancing 2΄‐OMe at the C3680 site of 28S rRNA. This rRNA modification increases the translation of SCD1, which in turn inhibits autophagy by modulating lipid peroxidation and mTOR activity.^108^ Moreover, SNORD42A is essential for acute myeloid leukaemia (AML) cell survival and proliferation, highly expressed in AML compared to normal cells. It directs 2΄‐OMe at uridine 116 of 18S rRNA, crucial for ribosomal protein translation. Knockout of SNORD42A impairs colony formation and proliferation, reducing cell size in leukaemia cells. This emphasises SNORD42A's key role in AML growth and survival through 18S rRNA modification.^109^ Notably, snoRNA‐mediated rRNA 2΄‐OMe may also play a tumour‐suppressive role. SNORA23 is downregulated and correlates with poor prognosis in HCC. Mechanistically, it inhibits HCC cell proliferation, migration, and invasion by reducing 2′‐O‐ribose methylation at cytidine4506 in 28S rRNA. This impairment of ribosome biogenesis by SNORA23, linked to the PI3K/Akt/mTOR pathway, decreases HCC growth, especially when combined with rapamycin.^110^

**TABLE 3:** snoRNAs involved in mediating rRNA modifications during cancer progression.

Cancer type	Regulators	rRNA type	Nucleotide	Modification type	Clinical significance	Ref
CRC	SNORD11B	18S rRNA	G509	2΄‐OMe	Promote cancer cell proliferation and invasion	107
NSCLC	SNORD88C	28S rRNA	C3680	2΄‐OMe	Promote NSCLC proliferation and metastasis	108
Acute myeloid leukaemia	SNORD42A	18S rRNA	U116	2΄‐OMe	Promote the translation of ribosomal proteins	109
HCC	SNORA23	28S rRNA	C4506	2΄‐OMe	Inhibit HCC cell proliferation, migration, and invasion	110
CRC	SNORD12C SNORD78	28S rRNA	Gm3878 Gm4593	2΄‐OMe	Contribute to CRC progression	112
Ovarian cancer	SNORA81	28S rRNA	U4606	Ψ	Decrease cell proliferation and migration	114
GCLC	SNORA56	28S rRNA	U1664	Ψ	Enhance the translation of GCLC	115
Breast cancer	SNORA67	18S rRNA	U1445	Ψ	Enhance ribosomal function and translational efficiency	116
HCC	SNORA24	18S rRNA	U609 U863	Ψ	Stable dynamics and reduced translational errors	117

NOP58 is a core protein in the assembly of box C/D snoRNPs, which are responsible for guiding the 2΄‐OMe of rRNA.^111^ Interestingly, ncRNAs can regulate rRNA 2΄‐OMe by affecting the expression of NOP58. For instance, the lncRNA ZFAS1 regulates NOP58, affecting rRNA 2΄‐OMe at specific sites—Gm3878 and Gm4593—via snoRNAs SNORD12C and SNORD78. This pathway contributes to CRC progression, with ZFAS1 knockdown leading to reduced cell proliferation and migration, and increased apoptosis.^112^ Similarly, MIR4435‐2HG, identified as an oncofetal lncRNA in hepatocellular carcinoma (HCC), is associated with poor prognosis and promotes stem‐cell properties and tumourigenesis. It binds NOP58 and IGF2BP1, with IGF2BP1 upregulating MIR4435‐2HG expression through m^6^A modification. This interaction stabilises NOP58, leading to increased rRNA 2΄‐OMe and enhanced IRES‐dependent oncogene translation, thereby playing a crucial role in HCC cell stemness and proliferation.^113^

### 7.4 snoRNA‐mediated rRNA pseudouridylation

The role of snoRNA‐mediated rRNA pseudouridylation in cancer is highlighted by a specific H/ACA snoRNA signature, including SNORA81, SNORA19, and SNORA56, which is pivotal in rRNA modification. This signature distinguishes between high‐grade serous ovarian carcinoma, serous borderline tumour, and normal tissues. Particularly, SNORA81 knockdown reduces rRNA pseudouridylation, leading to decreased cell proliferation and migration, thereby highlighting snoRNAs' influence on tumour aggressiveness through rRNA modification.^114^ Moreover, SNORA56, elevated in CRC tissues and plasma, correlates with CRC prognosis. Its deficiency hinders CRC cell proliferation and triggers ferroptosis, leading to reduced tumour growth. Mechanistically, SNORA56 facilitates psudouridylation of 28S rRNA at U1664, enhancing the translation of glutamate cysteine ligase, a key enzyme in glutathione biosynthesis that inhibits ferroptosis by preventing lipid peroxidation.^115^ In breast cancer, elevated dyskerin levels correlate with poor prognosis and aggressive tumour characteristics. Overexpression of DKC1 increases the expression specific snoRNAs, including SNORA67, leading to increased U1445 Ψ modification on 18S rRNA. This modification enhances ribosomal function and translational efficiency, indicating dyskerin contributes to the neoplastic phenotype in breast cancer through targeted rRNA modification.^116^ Interestingly, the loss of snoRNA‐mediated rRNA psudouridylation might promote cancer development. For instance, SNORA24 guides pseudouridine modifications in 18S rRNA and influences RAS‐induced senescence. Its loss, combined with RASG12V, accelerates liver cancer development in mice, mirroring human hepatocellular carcinoma. Low SNORA24 in human liver cancers is linked to worse prognosis and increased lipid content. Ribosomes lacking SNORA24's modifications on 18S rRNA exhibit disrupted dynamics and increased translational errors, highlighting the importance of snoRNA‐regulated rRNA modifications in cancer progression.^117^

### 7.5 Other important regulators for rRNA modifications

**FIGURE 7:**
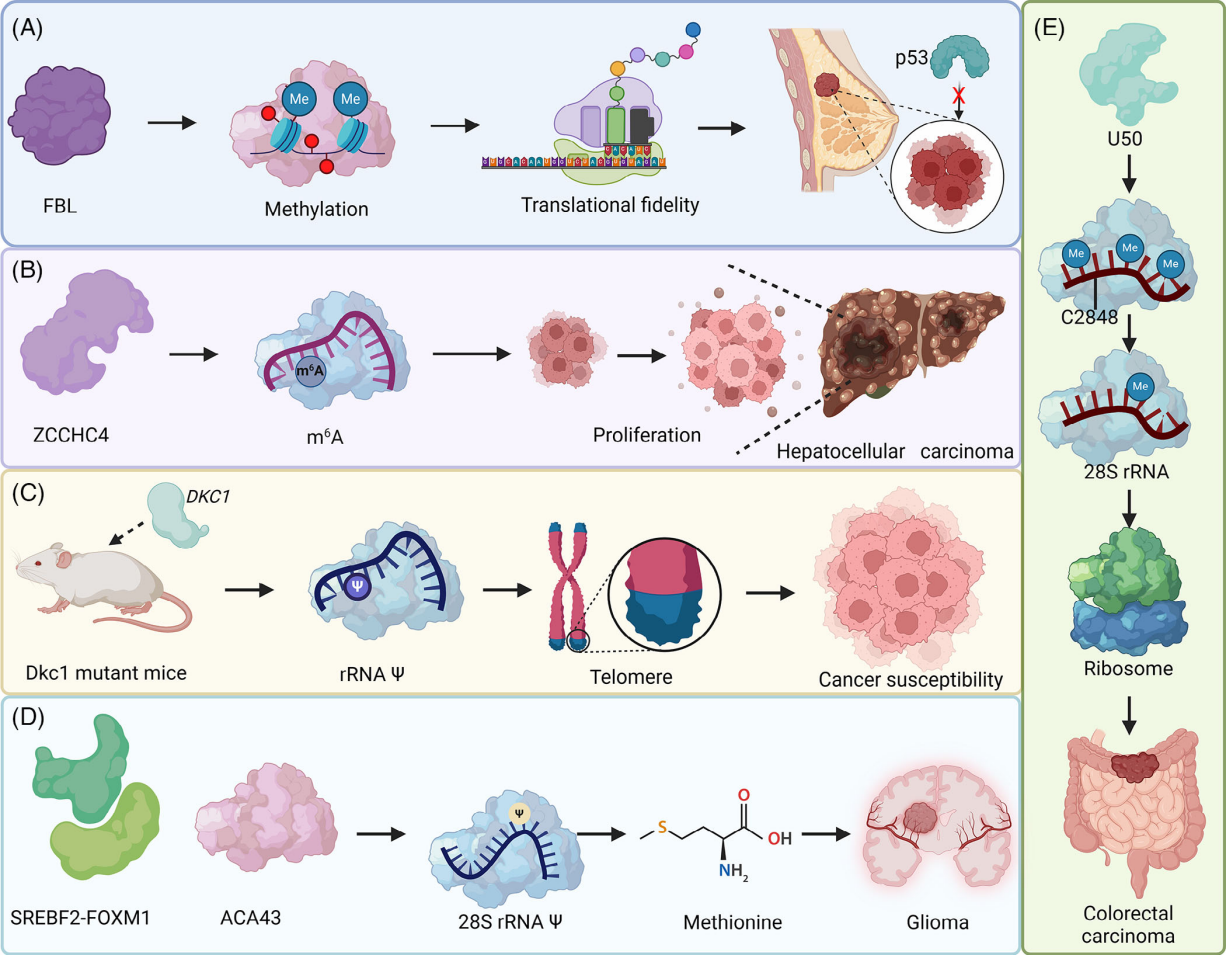
Additional key regulators of rRNA modifications in cancer. A. Overexpression of FBL in p53‐deficient cancer cells leads to altered rRNA methylation patterns, significantly impacting breast cancer progression. B. The loss of ZCCHC4 and the associated m^6^A_4220_ modification in 28S rRNA results in reduced global translation and impairs HCC growth. C. In Dkc1 mutant mice, early disruptions in rRNA psudouridylation precede DC symptoms, suggesting ribosome dysfunction as an initiator of DC. Telomere shortening in later generations contributes to DC progression and potential cancer susceptibility. D. Moreover, the SREBF2‐FOXM1 axis and ACA43 are critical in modulating 28S rRNA Ψ in small nucleolar RNA, affecting the progression of glioma‐initiating cells. This highlights the vital role of genetic factors and rRNA modifications in various cancers. E. U50 down‐regulation in cancers like colon cancer leads to diminished C2848 methylation of 28S rRNA, compromising ribosome function and cell proliferation. Created with BioRender.com.

In addition to METTL5/TRMT112 complex, NSUN5 and snoRNAs, other key regulators also play significant roles in rRNA modifications and ribosome biogenesis, significantly influence cancer progression (Figure 7). For instance, in p53‐inactivated cancer cells, overexpression of FBL alters rRNA methylation patterns, leading to impaired translational fidelity and increased IRES‐dependent initiation of key cancer genes. In addition, high FBL levels are linked to poor prognosis in breast cancer, highlighting p53's role in safeguarding ribosome quality and its impact on cancer progression.^118^ In addition, U50 is implicated in cancer development, with notable down‐regulation in colon cancer and other types. This reduction leads to less C2848 methylation in 28S rRNA, affecting ribosome function and cellular proliferation. Additionally, U50 influences ribosome efficiency in IRES‐mediated translation, highlighting the significance of specific rRNA methylation in ribosome activity.^119^ Moreover, knockout of ZCCHC4 leads to the loss of m^6^A_4220_ modification in 28S rRNA, resulting in reduced global translation and inhibited cell proliferation. Additionally, overexpression of ZCCHC4 in HCC suggests its role in cancer progression, as its knockout in a mouse model significantly decreases tumour size.^120^ Interestingly, in Dkc1 mutant mice, early rRNA psudouridylation impairments precede DC symptoms, indicating that ribosome dysfunction initiates DC. Telomere shortening, evident in later generations, contributes to DC's progression and potentially to its associated cancer susceptibility.^121^ Notably, methionine depletion in glioma‐initiating cells (GICs) leads to decreased cell proliferation and increased death, influenced by changes in cholesterol biosynthesis and rRNA modification. This involves down‐regulation of SREBF2‐FOXM1 and ACA43, a small nucleolar RNA guiding 28S rRNA psudouridylation, crucial for translation. These findings suggest that targeting methionine metabolism and rRNA modifications could be a potential strategy for controlling GICs in cancer therapy.^122^

## 8 THE POTENTIAL OF LEVERAGING RRNA MODIFICATION FOR GUIDING TUMOUR TREATMENT

As rRNA modifications play a pivotal role in the regulation of protein synthesis and cellular function, targeting these modifications presents an opportunity for developing novel cancer treatments. Drugs that modulate the activity of enzymes responsible for rRNA methylation or psudouridylation could selectively impact the translational machinery of cancer cells, sparing normal cells and reducing side effects. Additionally, rRNA modification patterns could serve as biomarkers for the prediction of treatment responses in cancer patients. This approach would enable more personalised and effective treatments, as therapies could be designed to target the unique ribosomal alterations present in an individual's tumour.^67^ For instance, high‐grade diffuse gliomas (HGGs) show distinct ribosome biology based on isocitrate dehydrogenase (IDH) status, with IDHwt glioblastomas having significant rRNA 2′O‐ribose methylation changes and IDHmut HGGs overexpressing ribosome biogenesis factors. Additionally, IDHmut HGGs exhibit higher sensitivity to RNA Pol I inhibitor CX5461 compared to IDHwt. These findings highlight ribosomal modifications as potential targets in HGG treatment, linked to IDH mutational status.^123^ Additionally, in colorectal carcinoma (CRC) and over 22 other cancer types, a notable single‐nucleotide variation in 18S rRNA at nucleotide 1248.U impacts the ancient m^1^acp^3^ Ψ modification. This variation, observed in up to 45.9% of CRC patients, leads to reduced m^1^acp^3^ Ψ levels in a subset of CRC tumours, creating a distinct class of “onco‐ribosomes.” These onco‐ribosomes, characterised by a translational profile rich in ribosomal proteins, contrast with normal tissues and suggest a new avenue for targeted cancer therapy.^124^ Moreover, hypomethylated sites like SSU‐C1440 in the small ribosomal subunit emerge as potential therapeutic targets in DLBCL, highlighting the importance of rRNA modifications in cancer development and treatment.^92^

## 9 DISCUSSION AND PROSPECTS

Cancer cells alter their protein synthesis patterns and accelerate the cell cycle to promote tumour growth, invasion, and spread. Specific rRNA modifications, like methylation and pseudouridylation, regulate intracellular translation mechanisms, influencing the molecular processes of tumourigenesis. Dynamic changes in rRNA modifications allow cancer cells to adjust their protein synthesis in varying microenvironments, thereby enhancing tumour adaptability and survival against treatments. While there is some understanding of the role of rRNA modifications in normal and cancerous cellular physiology, the precise mapping of modification sites, understanding specific regulatory mechanisms, and the broader impact on cancer development are not fully elucidated. In‐depth research into rRNA modifications could not only enhance our understanding of tumour biology but also provide crucial insights for developing new strategies for disease prevention, diagnosis, and treatment. These findings hold the potential for clinical application, benefiting cancer patients.

The complex structures and rich modifications of many translation‐related RNAs may impair sequencing efficiency and accuracy, creating a significant gap in our understanding of the spatial and temporal characteristics of highly structured and heavily modified RNAs like rRNA. There is an urgent need for new sequencing technologies with higher efficiency, precision, and sensitivity to overcome this challenge. Further advancement in the mapping and quantification of RNA modifications is required. Existing methodologies, such as RiboMeth‐seq, have markedly advanced the accuracy of rRNA modification mapping.^125^, ^126^ However, they still face limitations in detecting rare or subtle modifications against a backdrop of abundant unmodified rRNA, especially in heterogeneous tumour samples where different cell types may exhibit different modification patterns. Third‐generation sequencing methods like nanopore sequencing technology (Oxford Nanopore Technologies, ONT) have emerged as ideal alternatives. ONT sequencing platforms directly sequence DNA or RNA by monitoring the change in electrical current as individual molecules pass through a nanopore embedded in a synthetic polymer membrane, potentially detecting modifications like m^6^A based on the different current produced by modified bases. Future advancements need to focus on enhancing detection capabilities, possibly through innovative sequencing technologies or developing new chemical probes that selectively bind to modified rRNAs.^127^

The dysregulation of rRNA modifications, along with the aberrant expression of various enzymes involved in these modifications (referred to as writers, erasers, and readers), is linked to poor prognosis, therapy resistance, and impaired anti‐tumour immunity in various types of cancer.^128^As a result, the analysis of rRNA modification patterns, the enzymes that modify them, and the specific modification sites holds potential as diagnostic, prognostic, and predictive biomarkers. However, in practical clinical settings, it has been noted that RNA modifying enzymes can show similar patterns of dysregulation across different cancer types, and in some cases, exhibit contrasting roles in different cancers. Consequently, using overall modification levels as biomarkers may not be effective. Instead, identifying specific modifications at particular sites within individual rRNA molecules could prove more informative.^129^, ^130^ Precise determination of modification changes in individual transcripts, coupled with the enhancement of large‐scale transcriptomic mapping in both diseased and healthy samples, is essential for establishing effective predictive and prognostic models and for the screening and validation of reliable biomarkers for clinical use.

Understanding the regulation and function of rRNA modifications is crucial for advancing therapeutic applications. RNA modifications have significantly contributed to the development of various therapeutic approaches, such as antisense oligonucleotides, RNA therapeutics, and small interfering RNA drugs.^131^ Modified mRNA vaccines, particularly those with Ψ‐modification or N1‐methyl‐Ψ‐modification, have demonstrated remarkable success in treating infectious diseases, and their adaptations for cancer treatment are currently in development. Recent progress has been made with the development and clinical introduction of small molecule inhibitors targeting FTO and ALKBH5. These inhibitors, which focus on m^6^A modifications, have shown potential in suppressing the expression of immune checkpoint genes and reducing immune escape, thereby inhibiting tumour growth in a range of cancers.^132^ Consequently, the development of targeted therapies that regulate specific rRNA modifications represents a promising and innovative field. Potential strategies may include the use of small molecule inhibitors aimed specifically at enzymes responsible for rRNA modifications, or antisense oligonucleotides designed to alter rRNA modification patterns. However, there is still a notable gap in the availability of small molecule inhibitors or oligonucleotides that target rRNA modification sites. Additionally, our comprehension of how rRNA modifications regulate tumour immune mechanisms remains limited. A more in‐depth analysis of how various rRNA modifications influence tumour immune evasion could lead to the identification of more effective therapeutic targets and drugs, potentially enhancing treatment outcomes for cancer patients.^133^, ^134^, ^135^

In the field of cancer treatment research, widespread changes in RNA modifications open up new therapeutic and diagnostic avenues. More than 170 RNA modifications have been discovered, but the functions of associated enzymes and regulatory proteins for many modifications have not yet been determined.^136^ This has fuelled research into key components of the RNA modification machinery and their role in tumour biology. Research in recent years has revealed the important role that rRNA modification regulators play in regulating cancer progression. These regulators affect rRNA fate through specific modifications, thereby affecting tumour‐related phenotypes such as cell proliferation, metastasis, and drug resistance.^137^ Therefore, identifying the critical targets of rRNA modification and understanding their regulatory mechanisms is crucial for enhancing the therapeutic potential of rRNA modifications in cancer treatment.

Several strategies have been employed in therapeutic research targeting RNA modifications in tumour biology, including the development of small molecule inhibitors that target abnormalities in RNA modification mechanisms.^138^ These inhibitors have shown potential therapeutic efficacy in clinical trials, particularly when combined with other treatment strategies, potentially leading to synergistic effects that enhance treatment efficiency.^139^ Furthermore, recent research indicates that RNA modification plays a crucial role in regulating immune cell function and anti‐tumour immune responses.^140^ The involvement of specific RNA modification reading proteins suggests that the same RNA modification may have different functions in various cellular environments. Therefore, a deep understanding of the intracellular regulatory mechanisms of RNA modification is particularly important. It has been reported that METTL5/TRMT112 and its mediated m^6^A modification of 18S rRNA 1832 site (m^6^A_1832_) are increased in NPC, promoting tumour occurrence and resistance to chemotherapy, suggesting that METTL5 targeted inhibition agent has the potential to act as a synergistic rRNA modification and mRNA modification agent to treat cancer.^102^ However, a major challenge is the lack of specific drugs targeting METTL5, which limits treatment options and may increase adverse effects. RNA‐based therapeutic strategies are utilising RNA modifications to enhance the stability and efficiency of therapeutic molecules. Notably, targeted modification of stop codons has shown therapeutic potential, particularly in correcting genetic diseases caused by premature stop codons.^141^ Through specific rRNA modifications, such as pseudouridylation, the readthrough of stop codons can be enhanced, offering new treatment directions. In‐depth research and development of therapeutic strategies targeting rRNA modifications can provide more effective methods for cancer treatment, overcoming the limitations of existing therapies and introducing new therapeutic prospects.

## 10 CONCLUSIONS

In summary, rRNA modifications have emerged as a significant area of focus in current research, particularly due to their distinct impact on cancer studies. These modifications play a vital role in regulating rRNA function and cellular processes. Importantly, they are central to the progression of diseases and the emergence of treatment resistance, offering new insights and approaches for cancer treatment. The potential of rRNA modifications is especially relevant in addressing challenges like tumour heterogeneity and drug resistance, marking a promising area in cancer therapy. Despite significant progress in this field, a deeper understanding of these modifications remains essential for future advancements. Future research might focus on developing more precise diagnostic tools, personalised treatment strategies, and exploring the intricate relationships between rRNA modifications and other epigenetic changes. Overall, rRNA modifications represent a transformative aspect of cancer therapy, positioning them as a crucial element in combating this complex disease.

## AUTHOR CONTRIBUTIONS

Li Cui and Xinyuan Zhao conceptualised the idea of the review. Li Cui, Jiarong Zheng, and Yunfan Lin prepared initial drafts of the manuscript. Li Cui, Xinyuan Zhao, Jiarong Zheng, and Yunfan Lin contributed to the writing, graph creation, and manuscript improvement. Ye Lu, Yucheng Zheng, Pei Lin, and Bing Guo contributed to the creation of graphs and tables. All authors reviewed the manuscript and approved the final version of this manuscript.

## CONFLICT OF INTEREST STATEMENT

The authors declare no conflicts of interest.

## ETHICAL APPROVAL

Not applicable.

## Data Availability

Not applicable.
